# Enhancement in Mechanical and Electrical Properties of Polypropylene Using Graphene Oxide Grafted with End-Functionalized Polypropylene

**DOI:** 10.3390/ma9040240

**Published:** 2016-03-29

**Authors:** Patchanee Chammingkwan, Katsuhiko Matsushita, Toshiaki Taniike, Minoru Terano

**Affiliations:** Japan Advanced Institute of Science and Technology, 1-1 Asahidai, Nomi, Ishikawa 923-1292, Japan; chamming@jaist.ac.jp (P.C.); k.matsushita1989@gmail.com (K.M.); taniike@jaist.ac.jp (T.T.)

**Keywords:** nanocomposites, polypropylene, end-functionalization, grafting, graphene oxide

## Abstract

Terminally hydroxylated polypropylene (PP) synthesized by a chain transfer method was grafted to graphene oxide (GO) at the chain end. Thus obtained PP-modified GO (PP-GO) was melt mixed with PP without the use of a compatibilizer to prepare PP/GO nanocomposites. Mechanical and electrical properties of the resultant nanocomposites and reference samples that contained graphite nanoplatelets, partially reduced GO, or fully reduced GO were examined. The best improvement in the tensile strength was obtained using PP-GO at 1.0 wt %. The inclusion of PP-GO also led to the highest electrical conductivity, in spite of the incomplete reduction. These observations pointed out that terminally hydroxylated PP covalently grafted to GO prevented GO layers from re-stacking and agglomeration during melt mixing, affording improved dispersion as well as stronger interfacial bonding between the matrix and GO.

## 1. Introduction

Polymer nanocomposites are a class of hybrid materials in which nano-sized fillers are highly dispersed in polymer matrices. Compared with conventional polymer composites, nanocomposites often lead to remarkable reinforcement of physical properties of polymer and/or incorporation of novel functionality of nanomaterials. Nano-sized fillers offer a much greater interfacial area for effective load transfer to matrices. Moreover, a significantly enhanced particle density in polymer matrices enables the formation of percolation networks at a much smaller loading [1]. As growing need on stronger, lighter, more versatile, and less expensive materials with an ease of processing, polypropylene (PP) nanocomposites offer considerable advantages to satisfy these requirements.

The *sp*^2^-hydridized carbon atoms arranged in a honeycomb structure, termed as graphene, have attracted increasing attention since the first successful isolation of a single layer in 2004 [2]. Due to its extraordinary properties, such as thermal conductivity of 4.84–5.30 × 10^3^ W/mK [3], high specific surface area of 2630 m^2^/g [4], electrical conductivity of 7200 S/m [5], excellent mechanical strength of 130 GPa in the intrinsic fracture strength, and 1 TPa in the modulus [6], graphene has rapidly emerged as a material of choice among nano-sized fillers in polymer nanocomposites. Due to its extremely large aspect ratio, the antistatic criterion could be satisfied at a much lower percolation threshold [7,8]. Mechanical properties could be enhanced at maximum over 100% compared with pristine polymer at a small loading [9].

Despite the potential advantages, the dispersion of graphene in polymer matrices still remains challenging. Generally, single-layer graphene can be easily re-stacked upon drying due to the attractive van der Waals force between layers. The solution mixing is the best way to homogeneously mix graphene with polymer matrices. However, the solution blending is not economically viable and hardly applied to a polymer, such as PP, which only dissolves in specific solvents. Likewise, a few to several layers of graphene in the dried form are blended with PP in melt mixing, in which the performance of resultant nanocomposites greatly depends on the dispersion state in polymer matrices in addition to the aspect ratio of graphene layers and interfacial bonding with polymer matrices [10,11,12]. Since simple melt mixing often leads to unsatisfactory improvement of properties (due to the re-stacking of graphene layers, as well as their poor dispersion); an additional strategy is always required. Solid-state shear pulverization was reported to provide an excellent blending to enable *in-situ* exfoliation of graphite, which resulted in significant enhancement of Young’s modulus and yield strength by about 100% and 60% above pristine PP, respectively [13]. Another successful way is to blend PP latex with graphene oxide (GO) in water, followed by reduction and melt mixing with pristine PP. The PP latex inserts between GO layers and prevents their re-stacking upon drying and reduction [14,15,16]. Chemical functionalization has been also extensively studied, most of which have combined chemical functionalization of GO and the co-addition of maleic anhydride-grafted PP (PPMA) [17,18,19,20]. For example, hexamethylene diamine-modified GO was melt mixed with PP together with PPMA. They reported that PPMA was *in-situ* grafted to GO through amide linkages, leading to 14% of tensile strength improvement as compared to 6% for unmodified GO [18].

Recently, we have fabricated PP/PP-grafted SiO_2_ (PP-SiO_2_) nanocomposites, in which end-functionalized PP chains were grafted to SiO_2_ nanoparticles. It was found that the inclusion of PP-SiO_2_ enabled a 30% improvement in the tensile strength, which is unexpectedly large for spherical nanoparticles [21]. Such a large improvement was attributed to a physical cross-linkage structure through co-crystallization between matrix and grafted PP chains. It has been reported that polymer chains grafted at their side chains (e.g., in the case of PPMA) tend to wrap nanoparticles and form a soft layer on the surfaces, thus hampering mechanical reinforcement. On the other hand, polymer chains grafted at their chain ends form brush morphology, which endow superior reinforcement through entanglement, inter-diffusion, and co-crystallization mechanisms [22,23]. In this work, PP/graphene nanocomposites were prepared using GO modified with terminally hydroxylated PP, and their mechanical and electrical properties were evaluated.

## 2. Results and Discussion

### 2.1. Preparation of GO and rGO

**Figure 1:**
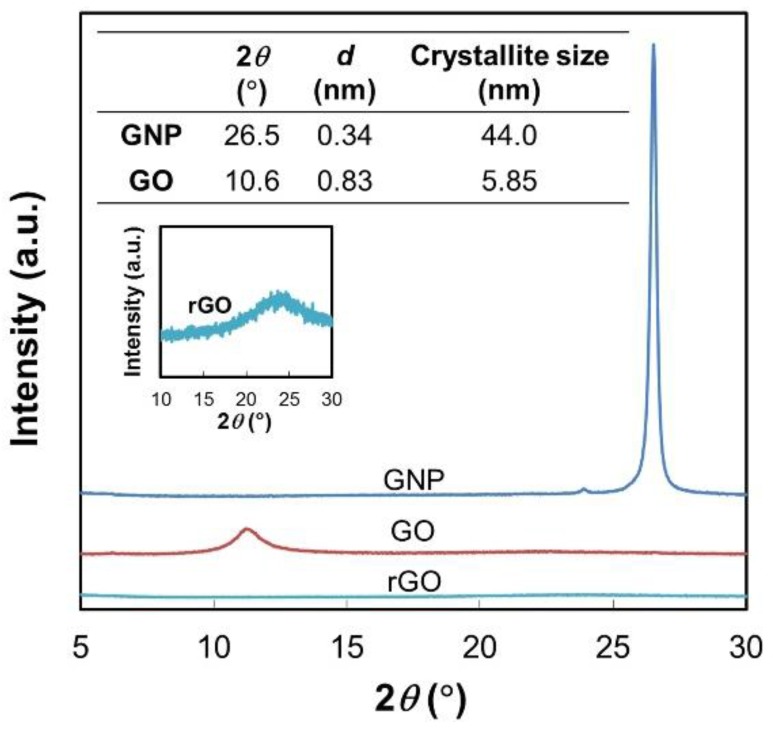
X-ray diffraction patterns of graphite nanoplatelets (GNP), graphene oxide (GO), and reduced GO (rGO) samples.

GO was prepared from graphite nanoplatelets (GNP) using a modified Hummer’s method [24,25,26,27]. After sonication, the sample was dried at 20 °C *in vacuo*. Reduced GO (rGO) was prepared by the thermal reduction of GO at 1050 °C. Figure 1 shows the diffraction patterns of all the samples. A peak at 2θ = 26.5° of GNP corresponds to the (002) plane, typically observed for graphitic carbon [11,28]. The *d*-spacing between layers calculated from the Bragg’s law [29] and the crystallite size calculated from the Scherrer equation [30] were 0.34 nm and 44.0 nm, respectively. After oxidation, the diffraction pattern of GO exhibits a peak at 2θ = 10.6°. The broadening of the peak compared to that of GNP indicated a decrease in the crystallite size and/or a less ordered structure. The *d*-spacing and the crystallite size were calculated as 0.83 nm and 5.85 nm, respectively. The disappearance of the peak at 26.5° and the increase of the *d*-spacing from the original GNP pointed out that the van der Waals interaction between layers became looser due to the intercalation of oxygen-containing functional groups [11]. After the thermal reduction, the (002) diffraction at 10.6° disappeared, while a very dispersive peak as an indicative of disordered graphitic structure was found around 24.6°. This is in accordance with literature, where the thermal treatment at high temperature (especially above 200 °C) caused the drastic cleavage of H_2_O and oxygen-containing functional groups from GO layers and the exfoliation of graphene [31,32,33].

**Figure 2:**
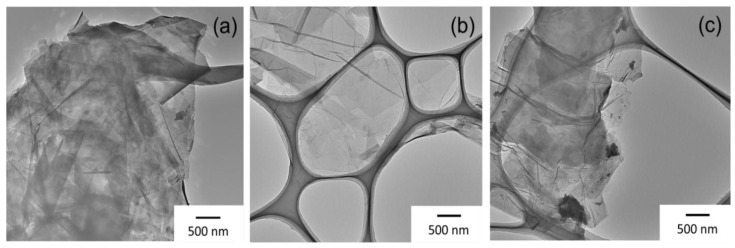
TEM images of (**a**) GNP; (**b**) GO; and (**c**) rGO samples.

A TEM image in Figure 2a shows that GNP was composed by a number of layers stacked with each other. Sheets with higher transparency were observed for GO (Figure 2b), indicating a significant exfoliation. After the thermal reduction, thick sheets were observed for rGO. This might be caused by the poor dispersibility in solvent during the preparation of a TEM grid.

**Figure 3:**
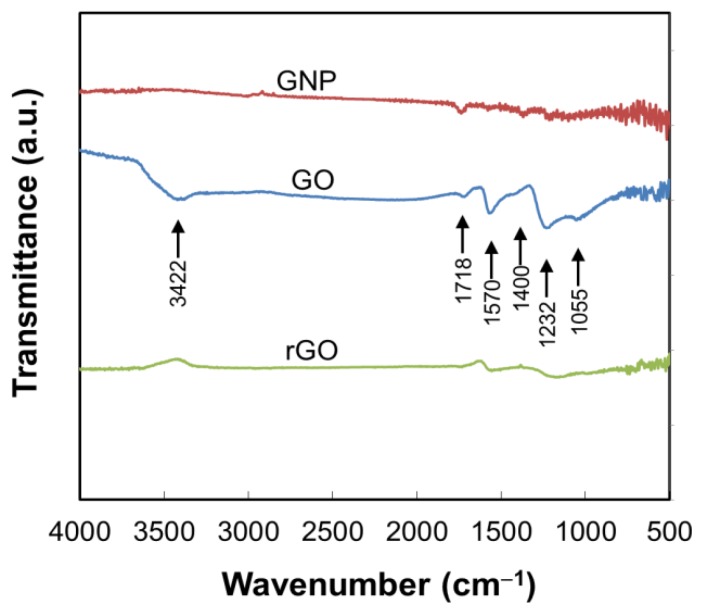
FTIR spectra of GNP, GO, and rGO samples.

The chemical structure of oxidized and reduced samples was confirmed by FTIR (Figure 3). It can be seen that the spectrum of original GNP exhibited small peaks at 1718 cm^−1^ for C=O stretching vibration and 1400 cm^−1^ for O−H in-plane deformation vibration of carboxylic groups [11,17,18,19], which are generally expected for GNP since the trigonal carbon bonds at the edges can easily react with ambient gas to form oxygen-containing functional groups and remain as impurities. The spectrum of GO exhibits the presence of various types of oxygen-containing functional groups. In addition to the vibration of carboxylic groups, the main peaks at 1232 cm^−1^ and 1055 cm^−1^ correspond to C−O stretching vibration of epoxy and alkoxy groups, respectively. The broad band at 3422 cm^−1^ and the peak at 1570 cm^−1^ are respectively attributed to O−H stretching vibration of hydroxyl groups [11,17,18,19] and C=C vibration from the aromatic ring [34]. After the thermal reduction at high temperature, most of these oxygen-containing functional groups were eliminated [31].

### 2.2. Synthesis of PP-OH

**Figure 4:**
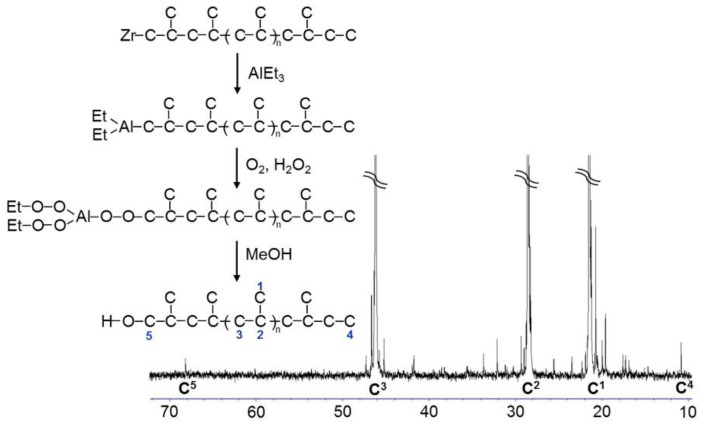
Synthesis scheme and ^13^C NMR spectrum of hydroxylated polypropylene (PP-OH).

Terminally hydroxylated PP (PP-OH) was prepared by a chain transfer method in propylene polymerization using triethylaluminum (TEA) as a chain transfer agent. *rac*-Ethylenebis(1-indenyl)zirconium dichloride (EBIZrCl_2_) was selected as the catalyst since its bulky ligand suppresses the β-H elimination. In such a circumstance, the growing chain at the metal centre predominantly transfers to Al of TEA, forming Al-terminated PP chains [35]. The obtained polymer was then converted to PP-OH by O_2_ insertion into the Al-R bonds, followed by hydrolysis (Figure 4). ^13^C NMR of PP-OH shows the peak of methylene carbon related to hydroxyl end groups (C^5^) at 68.5 ppm and the peak of methyl terminus as the chain head (C^4^) at 11.2 ppm, indicating the successful terminal hydroxylation of PP. The *mmmm*, *M_n_* and the percentage of end-functionalization [21] were 92 mol %, 1.2 × 10^4^ g/mol and 80 mol %, respectively.

### 2.3. Preparation and Characterization of PP Nanocomposites

**Figure 5:**
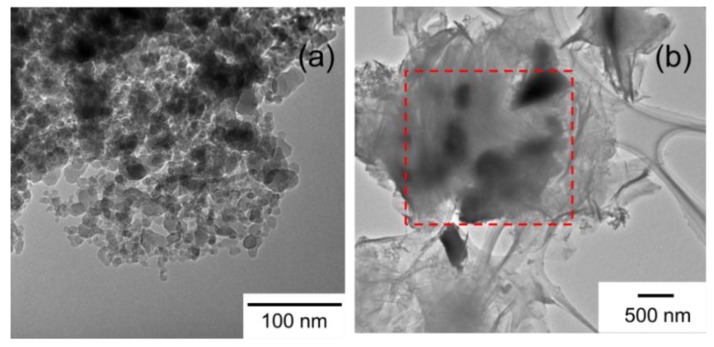
TEM images of (**a**) PP-OH; and (**b**) PP-modified graphene oxide (PP-GO) powder.

The modification of GO by PP-OH was attempted by heating PP-OH with GO in tetradecane at 200 °C. The resultant powder was washed repeatedly by hot filtration to obtain PP-modified GO (PP-GO). Figure 5a,b shows TEM images of the PP-OH and PP-GO powder. It was evidenced that GO sheets became partially less transparent compared to untreated GO (see Figure 2b) due to the existence of a polymer layer covering GO surfaces as marked in the dash square. The morphology of the polymer layer was totally different from that of the original PP-OH powder, since the treatment in tetradecane and hot filtration using ODCB dissolve PP-OH. Three scenarios could be suggested for the existence of the polymer layer: (i) the remaining of unwashed PP-OH; (ii) non-covalent interaction of PP-OH to GO; and (iii) covalent grafting of PP-OH to functional groups of GO. The former two scenarios were unlikely considering repetitive washing and the hydrophilic nature of partially reduced GO.

**Figure 6:**
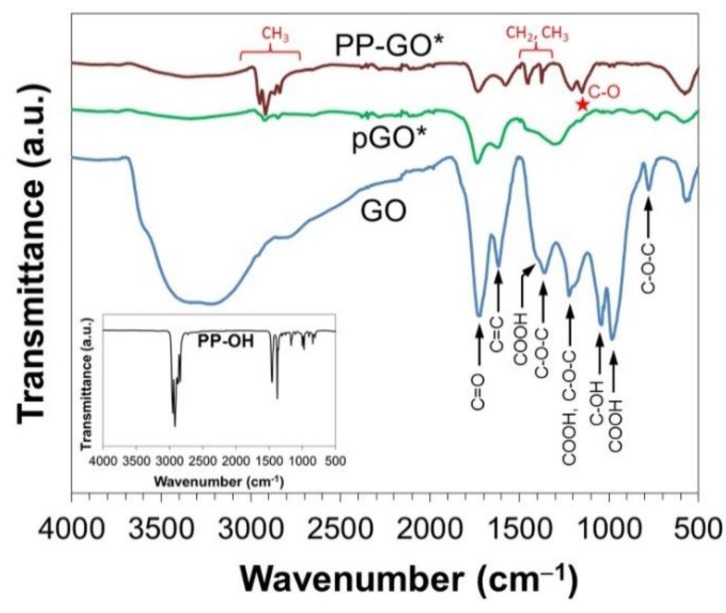
ATR-IR spectra of GO, pGO*, and PP-GO* samples.

In order to prove the existence of covalent grafting, GO was treated in tetradecane at 200 °C in the presence and absence of PP-OH without repetitive hot filtration, respectively denoted as PP-GO* and pGO*. After the removal of solvent and drying at room temperature, the chemical change near the surface was examined by attenuated total reflectance infrared spectroscopy (ATR-IR). As can be seen in Figure 6, the oxygen-containing functional groups of original GO, including those from carboxyl (COOH) at 1726, 1400, and 979 cm^−1^ [17,31,36], pyrene-like ether at 1357 cm^−1^ [37], carboxyl and epoxide at 1220 cm^−1^, alkoxide at 1040 cm^−1^ [20], and hydroxyl such as those from phenol (C–OH), COOH, and H_2_O at around 2855–3680 cm^−1^ and 1000–1060 cm^−1^ [38], were significantly reduced after the thermal treatment for both of the samples. Nonetheless, the ATR-IR spectra of PP-OH* and pGO* were significantly different: C–H vibration in the region of 1370–1460 cm^−1^ and 2800–2953 cm^−1^, attributed to PP-OH (shown as inset), was observed for PP-GO*. Small broad peaks of C–H vibration around 2800–2953 cm^−1^ in pGO* originated from the residual tetradecane solvent. The peaks around 1357–1400 cm^−1^ for pyrene-like ether and O–H bending of carboxyl totally disappeared for PP-GO* as compared to pGO*, and the peak of C–O stretching vibration of carboxyl and epoxide was shifted to 1207 cm^−1^ as compared to the original GO. Most interestingly, a new peak, which belongs to C–O stretching vibration of ester, appeared at 1152 cm^−1^ [39] only for PP-GO*. All of these facts indicated the formation of covalent linkages between PP-OH and carboxyl groups of GO. Although the ether linkage through the grafting of PP-OH to hydroxyl or epoxy groups of GO was not evidenced due to overlapping of C–O (ether) vibration around 1000–1250 cm^−1^, at least the covalent grafting of PP-OH to carboxyl groups of GO was successfully confirmed.

**Figure 7:**
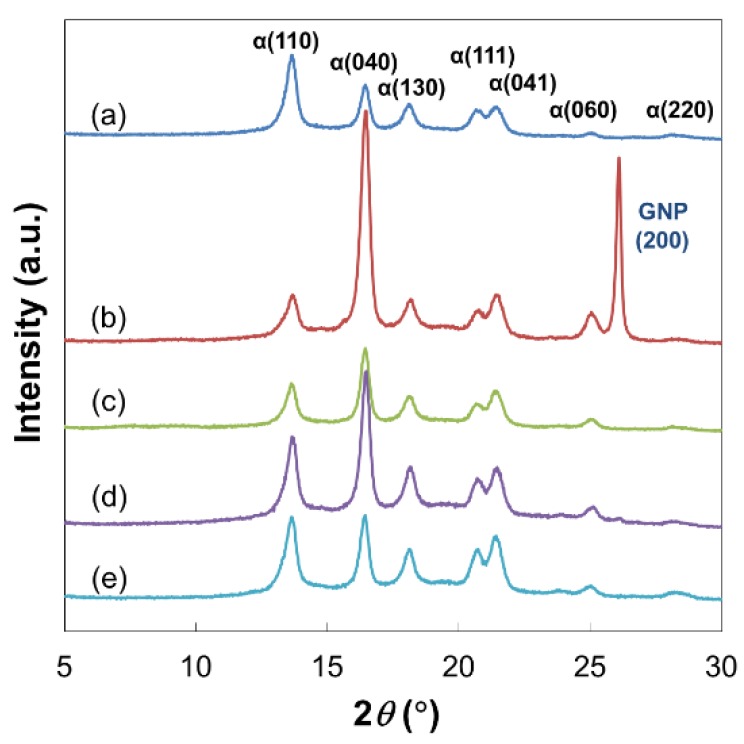
WAXD patterns of (**a**) pristine PP; (**b**) PP/GNP (3.0 wt %); (**c**) PP/rGO (3.0 wt %); (**d**) PP/pGO (3.0 wt %); and (**e**) PP/PP-GO (3.0 wt %) nanocomposites.

PP/PP-GO nanocomposites were prepared by melt mixing pristine PP with PP-GO at 1.0 or 3.0 wt %. PP/GNP and PP/rGO nanocomposites were also prepared as reference samples at the same filler contents. Since the modification of GO with PP-OH was performed at an elevated temperature, GO was partially reduced in the reaction. Therefore, additional reference nanocomposites with partially reduced GO (PP/pGO) were also prepared, in which pGO was treated in the same manner as PP-GO in the absence of PP-OH. The Wide-angle X-ray diffraction (WAXD) patterns of nanocomposites at 3.0 wt % of the filler loading are shown in Figure 7. Pristine PP exhibited the peaks at 2θ *=* 13.6°, 16.4°, 18.0°, 20.5°, 21.2°, 25.0°, and 27.9°, respectively, corresponding to (110), (040), (130), (111), (041), (060) and (220) planes of the α-form crystal of PP. The addition of GNP gave rise to a sharp peak at 26.1°, corresponding to the (002) diffraction of GNP. The absence of the same peak for PP/rGO, PP/pGO and PP/PP-GO nanocomposites confirmed the re-stacking-free dispersion in the PP matrix. The WAXD patterns of all the nanocomposites display the same α-form characteristics with the complete absence of the β-form, similarly to pristine PP. Nonetheless, the intensity of peaks at 2θ = 16.4° and 25.0°, respectively, corresponding to α(040) and α(060) planes, was significantly enhanced from that of pristine PP, and their extent was different among the nanocomposites. It has been reported that platelet fillers, including GNP [40] and talc [41], tend to align parallel to film surfaces in compression molding with its c*-axis perpendicular to the same surfaces. On the other hand, graphitic (001) planes have the crystallographic matching with the α-crystal form of PP with its b*-axis parallel to the c*-axis of the layers and offer a nucleating effect. Thus, orientation of graphitic layers causes the orientation of the PP crystal with its b*-axis perpendicular to the film surfaces, eventually resulting in the relative increase of the diffraction intensity for α(0*k*0) of PP [40]. From our results, all of the fillers exhibited a certain level of orientation of the PP crystal, as indicated by the increase of the α(040)/α(110) intensity ratio (Table 1). The most pronounced effect was found for GNP, followed by rGO > pGO > PP-GO, respectively. The smaller nucleation efficiency of rGO compared to GNP must be originated from oxidation/reduction damages to the sheets like wrinkles and buckling. In the case of pGO and PP-GO, the orientation degrees were smaller than those for GNP and rGO, plausibly because the remaining oxygen-containing functional groups deteriorated the nucleation efficiency. It should be noted that the orientation degree for pGO was significantly higher than that for PP-GO in spite of similar reduction extents. This result suggested that PP-OH covering GO surfaces might hinder the nucleation of matrix PP chains in a steric reason. In our previous work, the grafting of PP chains to SiO_2_ nanoparticles formed semi-dilute brush morphology and endowed the nucleation ability to SiO_2_ surfaces [21]. However, the least orientation degree for PP/PP-GO compared to the other samples indicated that this effect became less important for graphitic fillers, which equip a strong nucleation ability themselves. Rather, the presence of attached polymer chains and/or oxygen-containing functional groups prevented the nucleation.

The crystallization rate in isothermal crystallization was found to be in line with WAXD results (Table 2). All the nanocomposites showed enhanced crystallization rates compared to that of pristine PP. The crystallization rate increased by *ca.* three folds with the addition of 1.0 wt % of GNP and rGO, while by *ca.* two folds with the addition of 1.0 wt % of PP-GO. The increase in the filler content further enhanced the crystallization rate with marginal influences on the crystallinity and the melting temperature of the matrix.

**Figure 8:**
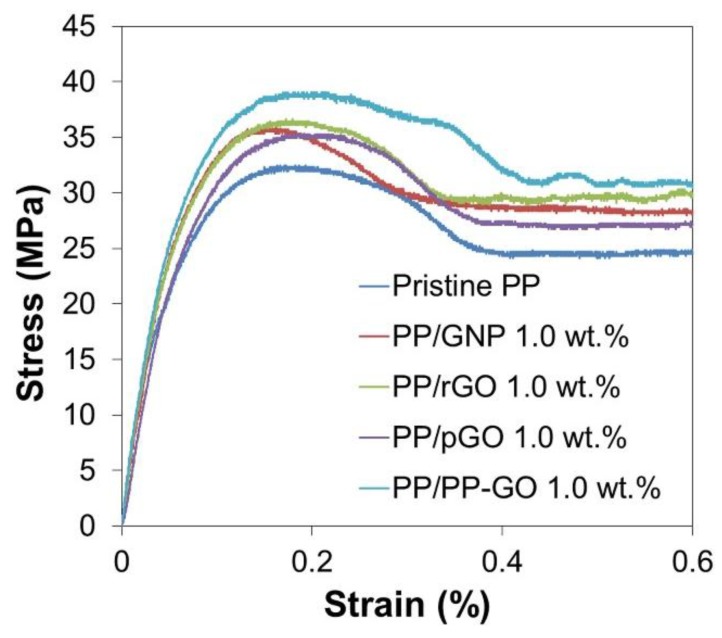
Tensile stress-strain curves of PP nanocomposites.

**Figure 9:**
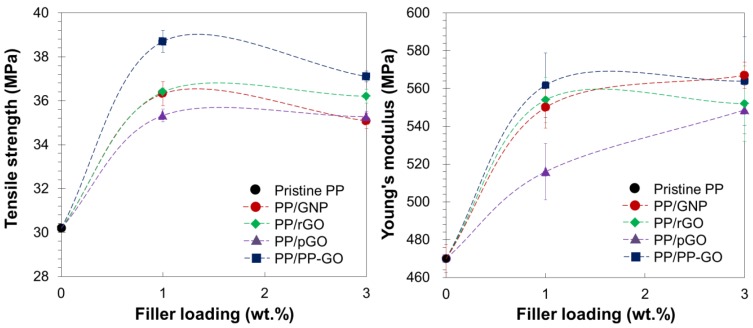
Tensile strength and Young’s modulus of PP nanocomposites at different filler loadings.

The uniaxial tensile test was conducted to evaluate the mechanical properties of the nanocomposites. Figure 8 exhibits the tensile stress-strain curves from the representative tests for nanocomposite samples (1.0 wt %), and the average properties from ten measurements are summarized in Table 3. The tensile strength and Young’s modulus are also plotted against the filler content in Figure 9. In general, mechanical properties of semi-crystalline polymer largely depend on the polymer crystallinity, while this dependency could be neglected in this study since all the nanocomposites had similar crystallinity to that of pristine PP. The addition of graphitic fillers at 1.0 wt % significantly enhanced the tensile strength and Young’s modulus for all the samples. On the other hand, slight decrements of the tensile strength were found when the filler content was increased to 3.0 wt %, plausibly due to deteriorated dispersion. The tensile strength of PP/rGO was slightly higher than that of PP/GNP, which was attributed to the increase in the layer density (in the matrix) due to the exfoliation. The presence of oxygen-containing functional groups deteriorated the mechanical reinforcement due to the poor compatibility and bonding with the matrix, as can be seen in the lowest tensile strength for PP/pGO. Nevertheless, PP/PP-GO exhibited the highest tensile strength compared to the other fillers with the enhancement of 28% from that of pristine PP at 1.0 wt %. This enhancement was comparably high to the reported +35% and +29% for the grafting of PPMA to diamine and amine-modified GO [17,18]. It must be noted that our nanocomposites were free from the compatibilizer as well as the organic modification of graphene layers. The highest tensile strength for PP/PP-GO indicated better dispersion as well as better interfacial interaction of PP-GO with the matrix, especially compared with rGO and pGO: the former represents the highest hydrophobicity (*i.e.*, the most compatible with PP), while the latter represents a similar thermal history. Likewise, the best reinforcement obtained for PP/PP-GO must be caused by the successful grafting of PP-OH to GO. The increase in the filler loading from 1.0 to 3.0 wt % tended to cause a drastic decrease of the elongation at break, which could be explained either by poor dispersion or by the reduction of the flexibility due to a barrier effect from platelet fillers that perturbs lamellae slippage, stretching, and orientation [19].

**Figure 10:**
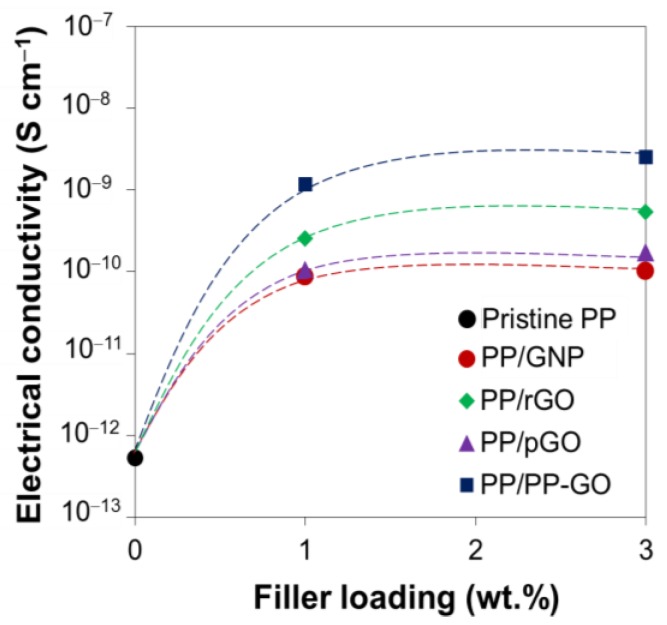
Electrical conductivity of PP nanocomposites at different filler loadings.

The electrical conductivity of the nanocomposites is plotted in Figure 10. The addition of all the types of graphitic fillers more or less increased the electrical conductivity to 1.0 wt %, while the further addition to 3.0 wt % hardly improved the conductivity. Similar saturation was also observed for the tensile strength. Both of these facts indicated the deterioration of the dispersion at 3.0 wt %. The increment of the electrical conductivity followed the order of pGO < GNP < rGO < PP-GO. In general, GO is an insulator due to the breakage of the pi-conjugated system, and the reduction recovers the original conductivity of graphene. For instance, the thermal reduction at 300 °C increases the conductivity around eight order of magnitude from pristine GO, and this restoration gradually approaches the original conductivity before oxidation with the increase of the thermal treatment temperature [33]. In this sense, the smaller conductivity for pGO as compared with GNP and rGO was quite reasonable. The higher conductivity for rGO as compared with GNP could also be explained by the larger particle density due to the exfoliation. Most surprisingly, PP-GO, which had a similar thermal history to pGO, showed the highest conductivity. This result again supported that the grafting of PP-OH to GO surfaces was successfully implemented and as a result, the dispersion of graphitic layers was improved in a way to overcome the lower reduction degree.

## 3. Experimental Section

### 3.1. Materials

Propylene monomer of research grade was donated by Japan Polypropylene Co. (Tokyo, Japan) and used as delivered. Modified methylaluminoxane (MMAO) as an activator and triethylaluminum (TEA) as a chain transfer agent were donated by Tosoh Finechem Co. (Shūnan, Japan). *rac*-Ethylenebis(1-indenyl)zirconium dichloride (EBIZrCl_2_) was purchased from Kanto Chemical. Co., Inc (Tokyo, Japan). Anhydrous toluene (Wako Pure Chemicals Industries, Ltd., Osako, Japan) was used after drying over a molecular sieve 4A. Tetradecace (Wako Pure Chemicals Industries, Ltd.) was dried over a molecular sieve 4A, followed by heating under N_2_ flow at 110 °C for 3 h. Graphite nanoplatelets (GNP, diameter of 5 μm and aspect ratio of 114, XG Sciences, Lansing, MI, USA) were dried under vacuum at 60 °C for 6 h prior to use. Concentrate sulfuric acid (conc. H_2_SO_4_, Kanto Chemical. Co., Inc.), potassium permanganate (KMnO_4_, Wako Pure Chemicals Industries, Ltd.) and 35% hydrogen peroxide aqueous solution (H_2_O_2_, Kanto Chemical. Co., Inc.) were used as received. Methanol, *o*-dichlorobenzene (ODCB), xylene, and *n*-hexane (Wako Pure Chemicals Industries, Ltd.) were used as washing solvents without purification. 3,5-Di-*tert*-butyl-4-hydroxytoluene (BHT, Wako Pure Chemicals Industries, Ltd.) was used as received. Octadecyl 3-(3,5-di-*tert*-butyl-4-hydroxyphenyl) propionate (AO-50) was donated by ADEKA Co., (Tokyo, Japan).

### 3.2. Synthesis of PP-OH

PP-OH was synthesized based on our previous report [21]. Propylene polymerization was conducted in a 500 mL round bottom flask equipped with an overhead motor stirrer rotating at 350 rpm. After sufficient N_2_ replacement, toluene as polymerization medium was charged into the flask and cooled down to 0 °C. 15.0 mmol of MMAO in toluene was introduced, followed by the addition of 5.0 µmol of EBIZrCl_2_ in toluene. 20 mmol of TEA was added as a chain transfer agent. The total volume of polymerization solvent was fixed at 300 mL. Propylene monomer was continuously introduced to keep the polymerization pressure at 1 atm. The polymerization was conducted at 0 °C under propylene flow for 1 h. Consequently, propylene was replaced by O_2_ and the polymer suspension was kept stirring under O_2_ bubbling for 1 h. Thereafter, 25.0 mL of 35% aqueous H_2_O_2_ was added, followed by the addition of 500 mL of methanol. Thus, obtained PP-OH was collected by filtration, washed with DI water, and purified by reprecipitation using xylene/methanol.

### 3.3. Preparation of GO, rGO, and pGO

Graphene oxide (GO) was prepared based on a modified Hummer’s method [24,25,26,27]. Next, 3.0 g of GNP was added to 69.0 mL of conc. H_2_SO_4_ pre-cooled at 0 °C under stirring at 350 rpm. Then 9.0 g of KMnO_4_ was slowly added, while keeping the temperature below 20 °C. The suspension was stirred at low temperature until the increment of temperature from reaction exotherm was fully settled, and then further stirred at room temperature for 30 min. Next, 69.0 mL of DI water pre-heated at 98 °C was added and the mixture was stirred for 15 min before adding 420 mL of DI water and 30 mL of 35% aqueous H_2_O_2_. The mixture was stirred at room temperature for 24 h before repetitive washing with DI water and centrifugation until the pH of the solution became neutral. GO powder was collected and dried *in vacuo* at 20 °C. Reduced graphene oxide (rGO) was prepared by the thermal treatment of GO at 1050 °C under N_2_ flow. Partially reduced graphene oxide (pGO) was prepared by heating GO power in tetradecane at 200 °C for 6 h, which mimicked the thermal history applied for the modification of GO with PP-OH.

### 3.4. Preparation of PP-GO

PP-OH and GO were dried under vacuum to remove physisorbed water. Next, 3.0 g of PP-OH and 1.0 g of GO were introduced into a round bottom flask. Then 300 mL of dehydrated tetradecane, containing 0.03 wt % of AO-50 with respect to PP-OH, was introduced and the suspension was heated at 200 °C for 6 h under stirring at 350 rpm. After filtration, the powder was repeatedly washed with methanol before drying at 60 °C *in vacuo* for 6 h. The ungrafted PP-OH was removed by repetitive hot filtration at 140 °C using ODCB containing BHT. Thus, obtained PP-modified GO (PP-GO) was finally washed with hexane and dried at 60 °C *in vacuo* for 6 h.

### 3.5. Preparation of PP Nanocomposites

PP nanocomposites were prepared by melt mixing of pristine PP with 1.0 or 3.0 wt % of PP-GO using a two-roll mixer. PP pellet (*M_w_* = 3.2 × 10^5^ g/mol, *M_w_*/*M_n_* = 4.2, *mmmm* = 98%) was firstly kneaded at 185 °C for 5 min in the presence of 0.1 wt % AO-50 as an anti-oxidant. After the addition of PP-GO, polymer was further kneaded at 185 °C for an additional 10 min. The product was then hot-pressed into a film with the thickness of 200 µm using the pressure of 20 MPa at 190 °C, followed by quenching at 100 °C for 5 min and 0 °C to obtain PP/PP-GO nanocomposite films. Reference samples were prepared using GNP, rGO, and pGO instead of PP-GO at the same loadings.

### 3.6. Characterization

Wide-angle X-ray diffraction (WAXD) patterns were recorded on Smart Lab (Rigaku, Japan) operated at 40 kV and 30 mA with CuKα radiation at the step size of 0.02° in the range of 2θ = 5°–30°. The crystallite size and *d*-spacing of GNP and GO were calculated based on the Scherrer equation and the Bragg’s law, respectively. Fourier transform infrared spectroscopy (FTIR, FT/IR-6100 JASCO, Tokyo, Japan) was used to confirm the formation and reduction of GO. The spectra were obtained with the number of scans of 24 at a resolution of 4 cm^−1^ in the range of 400–4000 cm^−1^. Attenuated total reflectance infrared spectroscopy (ATR-IR, Spectrum100, PerkinElmer, Waltham, MA, USA) was used to examine the chemical nature near the surface of treated GO. The spectra were obtained with the number of scans of 40 at a resolution of 4 cm^−1^ in the range of 400–4000 cm^−1^. Transmission electron microscope (TEM) images were obtained using a Hitachi H-7100 (Hitachi, Tokyo, Japan) operated at 100 kV. The molecular weight (*M*_n_) and % end-functionalization of PP-OH were measured by ^13^C NMR (400 MHz, Bruker, Pittsburgh, PA, United States) at 120 °C using 1,2,4-trichlorobenzene as diluent and 1,1,2,2-tetrachloroetane-*d*_2_ as internal lock and reference. The crystalline structure of PP nanocomposites was analyzed using WAXD. The measurements were performed in a reflection mode at room temperature. The melting temperature and isothermal crystallization behavior of nanocomposites were evaluated by a differential scanning calorimeter (DSC, Mettler Toledo DSC 822, Mettler Toledo, Columbus, OH, USA). The sample crystallinity was determined from the melting endotherm in the first heating cycle, where the sample was heated from 50 to 200 °C at the heating rate of 10 °C/min. Isothermal crystallization was investigated at 128 °C. The sample was kept at 200 °C for 5 min to erase the thermal history, and then cooled down to 128 °C at the cooling rate of 40 °C/min. Tensile properties of nanocomposites were obtained using an Abe Dat-100 tensile tester (Abecks Inc., Tokyo, Japan). The measurements were basically carried out according to ASTM D638 using a specimen of type IV with reduced dimension. The sample was die cut in to a dumbbell-shape with the overall length of 23 mm and the gage length of 5 mm. The test was conducted at a crosshead speed of 1.0 mm/min at room temperature. The tensile properties, such as the tensile strength and Young’s modulus, were determined as average values from ten measurements. The electrical conductivity of nanocomposites was evaluated from surface resistivity using a two-point probe method (R8340A, ADVANTEST, Tokyo, Japan) at 100 V under 20% RH.

## 4. Conclusions

PP/GO nanocomposites were prepared based on a polymer grafting strategy, where terminally hydroxylated PP synthesized by a chain transfer method was grafted to GO prior to melt mixing with the matrix PP. The increment of tensile strength of 28% over pristine polymer was achieved with the inclusion of PP-GO at 1.0 wt %. The enhancement was greater than those obtained for GNP, partially reduced GO, and fully reduced GO at the same loadings. The electrical conductivity also became the highest for PP-GO irrespective of its incomplete reduction. All of the results pointed out that terminally hydroxylated PP covalently grafted to GO prevented graphitic layers from re-stacking and agglomeration, and improved the interfacial interaction between the matrix and GO.

## Figures and Tables

**Table 1:** Intensity ratio of α(040)/α(110) for PP nanocomposites.

Sample	α(040)/α(110)
Pristine PP	0.66
PP/GNP (3.0 wt %)	4.31
PP/rGO (3.0 wt %)	1.93
PP/pGO (3.0 wt %)	1.68
PP/PP-GO (3.0 wt %)	1.02

**Table 2:** DSC results for PP nanocomposites.

Sample	*t_1/2_*^−1^ ^a^ (min^−^^1^)	*X_c_* ^b^ (%)	*T_m_* ^c^ (°C)
Pristine PP	0.18	47.9	164
PP/GNP (1.0 wt %)	0.68	47.1	161
PP/GNP (3.0 wt %)	0.97	46.0	161
PP/rGO (1.0 wt %)	0.74	47.6	162
PP/rGO (3.0 wt %)	1.03	46.5	163
PP/PP-GO (1.0 wt %)	0.35	46.5	159
PP/PP-GO (3.0 wt %)	0.40	47.1	162

^a^ *t_1/2_*: Half time of isothermal crystallization at 128 °C; ^b^ *X_c_*: Crystallinity of PP; ^c^ *T_m_*: Melting temperature.

**Table 3:** Tensile properties of PP nanocomposites.

Sample	Tensile Strength (MPa)	Young’s Modulus (MPa)	Elongation at Break (%)
Pristine PP	30.2 ± 0.4	470 ± 15	>300
PP/GNP (1.0 wt % )	36.3 ± 1.1	550 ± 22	>300
PP/GNP (3.0 wt %)	35.1 ± 0.7	567 ± 14	38 ± 8
PP/rGO (1.0 wt %)	36.4 ± 0.4	554 ± 24	>300
PP/rGO (3.0 wt %)	36.2 ± 0.6	552 ± 40	62 ± 14
PP/pGO (1.0 wt %)	35.3 ± 0.6	516 ± 29	>300
PP/pGO (3.0 wt %)	35.3 ± 0.5	549 ± 25	79 ± 12
PP/PP-GO (1.0 wt %)	38.7 ± 1.0	562 ± 34	>300
PP/PP-GO (3.0 wt % )	37.1 ± 0.5	564 ± 47	>300
